# CoA in Health and Disease

**DOI:** 10.3390/ijms23084371

**Published:** 2022-04-15

**Authors:** Pawel Dobrzyn

**Affiliations:** Laboratory of Molecular Medical Biochemistry, Nencki Institute of Experimental Biology, Polish Academy of Sciences, 02-093 Warsaw, Poland; p.dobrzyn@nencki.edu.pl

Coenzyme A (CoA) and its thioester derivatives are crucial components of numerous biosynthetic and degradative pathways of the cellular metabolism (including fatty acid synthesis and oxidation, the Krebs cycle, ketogenesis, cholesterol and acetylcholine biosynthesis, amino acid degradation, and neurotransmitter biosynthesis), post-translational modifications of proteins, and the regulation of gene expression. Consequently, unsurprising is the fact that the abnormal biosynthesis of CoA/CoA derivatives is associated with numerous pathologies, including diabetes, neurodegeneration, Reye’s syndrome, vitamin B12 deficiency, cardiac hypertrophy, and cancer. Therefore, the contents of CoA and its derivatives are strictly controlled by nutrients, hormones, metabolites, and cellular stresses [1]. This Special Issue of *IJMS* focuses on novel research and review articles encompassing multiple roles of CoA and its derivatives in extracellular and intracellular signaling functions in physiology and pathology.

The pathophysiological role of CoA is described in detail in a review by Czumaj et al. [2]. The authors mainly focused on subcellular CoA concentrations, the roles of CoA in synthesis and degradation processes, and protein modifications by reversible CoA binding to protein, considering also the role of CoA in the pathogenesis of neurodegenerative diseases, cancer, myopathies, and infectious diseases and the beneficial outcome of CoA in the treatment of hyperlipidemia.

Coenzyme A and acetyl-CoA are important regulators of the cellular energy metabolism. Shurubor et al. [3] evaluated the delayed effect of hepatotoxin thioacetamide (TAA) on CoA and acetyl-CoA concentrations in plasma, the liver, the kidneys, and the brain in rats. Their data indicated that even a single administration of TAA in rats is sufficient to alter the physiological balance of CoA and acetyl-CoA in plasma and tissues in rats for an extended period of time. This study highlighted that maintaining optimal levels of CoA in various tissues and organs under physiological and pathological conditions and understanding the possible mechanisms that affect cellular CoA levels can help normalize the function of the Krebs cycle in certain types of diseases.

Mutations of the pantothenate kinase 2 (*PANK2*) gene are a reason for pantothenate kinase-associated neurodegeneration (PKAN), the most common form of neurodegeneration associated with brain iron accumulation. Berti et al. [4] constructed and characterized a yeast model of PKAN. They deleted the *CAB1* gene and expressed pathological variants of *PANK2* in yeast. This model mimics the main phenotypes associated with human PKAN, namely, mitochondrial dysfunction, alterations of lipid metabolism, iron overload, and oxidative damage; thus, constituting a useful model to investigate PKAN pathogenesis and identify new therapeutic candidate molecules for the treatment of PKAN.

To gain new insights into the mechanisms that connect CoA metabolism, iron dyshomeostasis, and neurodegeneration, a new mammalian model of COASY protein-associated neurodegeneration (CoPAN) was created by Di Meo et al. [5]. A conditional neuronal-specific COASY knockout mouse consistently developed a severe early-onset neurological phenotype that was characterized by sensorimotor defects, dystonia-like movements, impairments in iron homeostasis, and mitochondrial dysfunction, leading to premature death. These two new research models presented above are related to human disorders and could likely contribute to elucidating the pathogenesis of these diseases and the development of new therapeutic approaches.

Stearoyl-CoA desaturase (SCD) is the rate-limiting enzyme that catalyzes the synthesis of monounsaturated fatty acids, mainly oleate and palmitoleate, which are used as substrates for the synthesis of triglycerides, wax esters, cholesterol esters, and phospholipids [6]. The available data indicate that monounsaturated fatty acids reduce key risk factors in metabolic syndrome. Dietary monounsaturated fatty acids promote a healthy blood lipid profile, lower blood pressure, and positively modify insulin sensitivity and glycemic control [7]. The review article by Revaut et al. [8] summarized our current knowledge of the molecular effects of specific classes of fatty acids (saturated and unsaturated) to better understand the impact of different diets (e.g., Western vs. Mediterranean) on inflammation based on a metabolic background. Based on positive effects of monounsaturated fatty acids abundant in the Mediterranean diet, recent data on the role of SCD1 activity in modulating saturated fatty acid-induced chronic inflammation were also discussed.

The current findings suggest that SCD1 is a very promising novel target for the treatment of insulin resistance and the preservation of proper β-cell function. The research article by Dobosz et al. [9] investigated the role of SCD1 in maintaining the DNA methylation profile in pancreatic β-cells. Their major finding was that SCD1 inhibition/gene silencing altered the distribution of -CH3 groups within chromosomes and elicited DNA hypomethylation in β-cells. These effects appeared to be mediated by the adenosine monophosphate-activated protein kinase/sirtuin 1-dependent downregulation of DNA-methyltransferase 1 (DNMT1). Overall, the results suggest that SCD1 protects β-cells against the loss of DNA methylation caused by lipid overload, suggesting a mechanism by which SCD1 regulates DNA methylation patterns in pancreatic islets and in type 2 diabetes-relevant tissues.

SCD1 is also an important player in the regulation of heart metabolism. *SCD1* deficiency reduces fatty acid uptake and utilization in the heart. The loss of *SCD1* decreases the expression of genes involved in fatty acid transport and lipogenesis in the heart, alongside a reduction in cardiac free fatty acid, diacylglycerol, triglyceride, and ceramide levels [6]. The study by Olichwier et al. [10] showed that a normal thyroid hormone metabolism is necessary to maintain the antisteatotic effect of *SCD1* downregulation in the heart and may be involved in the upregulation of energetic metabolism associated with *SCD1* deficiency.

*SCD2* is ubiquitously expressed, except in the adult liver, and is the predominant isoform in the brain [6]. O’Neill et al. [11] showed that bone mineral density is decreased in *SCD2*-deficient mice under high-fat feeding conditions and that SCD2 is not required for preadipocyte differentiation or the expression of the peroxisome proliferator-activated receptor γ in vivo, despite being required in vitro. Moreover, an inclusive review of SCD2 function in mouse development, metabolism, and various diseases (e.g., obesity, chronic kidney disease, Alzheimer’s disease, multiple sclerosis, and Parkinson’s disease) is provided in this article. Overall, the papers presented in this Special Issue of *IJMS* significantly increase our knowledge of the metabolic role of SCD.

A review by Abbassi et al. [12] provides a thorough description of recent developments around the highly conserved catalytic subunit Elp3 of the eukaryotic Elongator complex from bacteria, archaea, and eukaryotes. The authors mainly focused on available structural and biochemical details and connected these data to currently available clinical evidence.

Information regarding the regulation of CoA biosynthetic complex assembly in mammalian cells is presented by Baković et al. [13]. This interesting study presented the assembly of all five enzymes that drive CoA biosynthesis in HEK293 cells with stable Pank1β expression and in an A549 lung cancer cell line using an in situ proximity ligation assay. The authors showed that the association of CoA biosynthetic enzymes is strongly upregulated in response to serum starvation and oxidative stress, whereas insulin and growth factor signaling downregulated their assembly. This study is likely to enable further investigations of the mode of interaction, structural organization, regulation, and the subcellular localization of the potential CoA biosynthetic complex in mammalian cells.

In summary, the topic of this Special Issue highlights the important role of CoA and pathways that regulate its content and transformations in tissues and organs in regulating cellular signaling pathways in physiology and pathology. We hope that this Special Issue will attract further attention and interest among the scientific community and contribute to the design of further research on CoA function in pathology and physiology.
